# *Tupaia* GBP1 exploits autophagy to restrict herpes simplex virus type 1 infection

**DOI:** 10.1080/27694127.2022.2030506

**Published:** 2022-03-17

**Authors:** Tianle Gu, Yong-Gang Yao

**Affiliations:** a Key Laboratory of Animal Models and Human Disease Mechanisms of the Chinese Academy of Sciences & Yunnan Province, and KIZ-CUHK Joint Laboratory of Bioresources and Molecular Research in Common Diseases, Kunming Institute of Zoology, Kunming, Yunnan 650204, China; b Kunming College of Life Science, University of Chinese Academy of Sciences, Kunming, Yunnan 650204, China; c National Resource Center for Non-Human Primates, and National Research Facility for Phenotypic & Genetic Analysis of Model Animals (Primate Facility), Kunming Institute of Zoology, Chinese Academy of Sciences, Kunming, Yunnan 650107, China

**Keywords:** Autophagy, GBP1, HSV-1 infection, interferon, STING1, tree shrew

## Abstract

Macroautophagy/autophagy initiated by STING1 (stimulator of interferon response cGAMP interactor 1) is actively involved in viral infection; and it is known that interferon-inducible guanylate-binding proteins (GBPs) exploit autophagy to defend the host against bacterial infection. Here we showed that in the tree shrew (*Tupaia belangeri*), a close relative to primates, TbGBP1 (*T. belangeri* GBP1) interacts with TbSTING1 to initiate autophagy and inhibit herpes simplex virus type 1 (HSV-1) infection.

To fight against virus infection, eukaryotic organisms have evolved the interferon (IFN) system to induce hundreds of interferon-stimulated genes (ISGs) encoding putative antiviral factors. GBP1 (guanylate binding protein 1) is an ISG and belongs to the interferon-inducible guanylate-binding protein (GBP) family that is central to the cell-autonomous immunity in defending the host against microbial infections. Despite the role of GBPs in bacterial and protozoan infections being well documented, a lot of effort is still needed to understand GBP functions in response to viral infections. STING1 plays a critical role in fighting against viral infections via meditating the induction of IFNs and autophagy, but the regulation of STING1 in the initiation of autophagy has not been sufficiently determined.

By using the Chinese tree shrew (*Tupaia belangeri chinensis*), we previously found that overexpression of TbGBP1 inhibits HSV-1 production in the tree shrew primary renal cells (TSPRCs) and in the tree shrew renal cell line, TSR6. Moreover, knockout of TbGBP1 in TSR6 (TSR6-*TbGBP1*-KO) cells significantly promotes HSV-1 production, whereas overexpression of TbGBP1 in TSR6-*TbGBP1*-KO cells can damage the permissiveness of TSR6-*TbGBP1*-KO cells for HSV-1 and inhibits HSV-1 production. Autophagy is induced in tree shrew cells upon HSV-1 infection. We performed co-immunoprecipitation screening assays and found that TbGBP1 interacts with TbSTING1, but not with TbIFIH1/MDA5 (interferon induced with helicase C domain 1) and TbMAVS (mitochondrial antiviral signaling protein), which are all key immune factors in the antiviral pathway. Furthermore, we found that the interaction between TbGBP1 and TbSTING1 is mainly mediated by the GTPase domain of TbGBP1 and the cGAMP-binding domain of TbSTING1. STING1 has been reported to induce IFN production and autophagy to restrict viral infections. We found that TbGBP1 overexpression does not affect the interferon signaling and downstream expression of IFN-stimulated genes, such as *TbOAS1* (2’-5’ oligoadenylate synthetase 1) upon HSV-1 infection. Instead, the TbSQSTM1 (sequestosome 1) protein level is decreased and the LC3-II:LC3-I ratio is elevated in TbGBP1-overexpressing TSPRCs in response to HSV-1 infection, suggesting enhanced autophagy. Knockout of *TbGBP1* in TSR6 cells abolishes the autophagy induced by HSV-1 infection, which can be reversed by TbGBP1 overexpression. These observations indicated that TbGBP1 promotes autophagy but not IFN signaling upon HSV-1 infection.

In humans, STING1 induces autophagy by a direct interaction with SQSTM1 and LC3. We speculated that the TbGBP1 may participate in the interaction among TbSTING1, TbSQSTM1 and TbLC3. We found that overexpressed TbSTING1 does not interact with TbSQSTM1 or TbLC3, but overexpressed TbGBP1 interacts with both TbSQSTM1 and TbLC3. Consistently, overexpressed TbGBP1 colocalizes with overexpressed TbSQSTM1 and TbLC3 in TSPRCs. Moreover, when TbGBP1 and TbSTING1 are overexpressed together with TbSQSTM1 or TbLC3, we can observe an interaction of TbGBP1-TbSTING1-TbSQSTM1 and of TbGBP1-TbSTING1-TbLC3. Thus, TbGBP1 can bind to TbSTING1, TbSQSTM1 and TbLC3, and function as a scaffold to promote autophagy in response to HSV-1 infection (Fig. 1).

**Figure 1.**
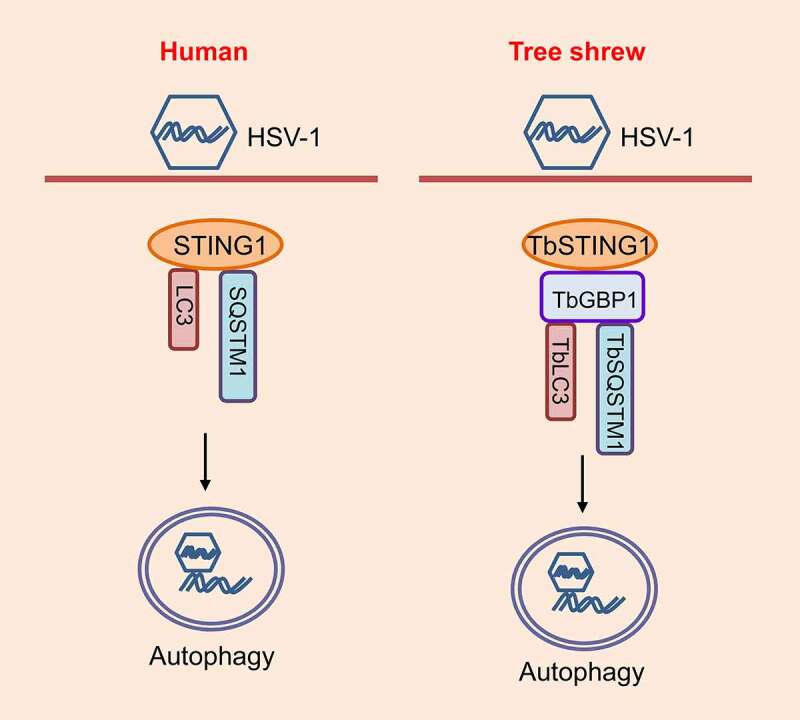
*Tupaia* GBP1 interacts with TbSTING1 to initiate autophagy and restricts HSV-1 infection. In contrast to human cells, TbSTING1 does not interact with TbSQSTM1 and TbLC3 in tree shrew cells. TbGBP1 acts as a scaffold for TbSTING1, TbSQSTM1 and TbLC3 interaction and promotes autophagy in response to HSV-1 infection.

With these results, we think that TbGBP1 controls HSV-1 infection by interacting with TbSTING1 and harnessing autophagy. We further examined the anti-HSV-1 function of TbGBP1 in the absence of TbSTING1 in TSR6 cells, to verify this speculation. The anti-HSV-1 effect of TbGBP1 is lost in TSR6 cells with *TbSTING1* knockout (TSR6-*TbSTING1*-KO). Overexpression of TbSTING1 in TSR6-*TbSTING1*-KO cells recovers the anti-HSV-1 capability of TbGBP1. Consistently, overexpression of a truncated TbSTING1 (TbSTING1-ΔCTT), which possesses the ability to induce autophagy but without type I IFN induction activity, also recovers the anti-HSV-1 activity of TbGBP1 in TSR6-*TbSTING1*-KO cells. These results indicated that the TbGBP1 interacts with TbSTING1 to control HSV-1 infection via autophagy [1].

The GBPs can be induced by many kinds of cytokines, such as IFNA, IFNB, IFNG, TNF/TNFα, and toll-like receptor (TLR) agonists, suggesting a functional diversity. GBPs can target to and disrupt the membrane-enveloped bacteria and parasites. For instance, GBP1 acts as a *bona fide* pattern recognition receptor for bacterial lipopolysaccharide to disrupt the integrity of bacterial outer membranes. In addition, GBPs targets to endosomes and lysosomes. Autophagy proteins have an active involvement in the membrane targeting of GBPs, but with different interaction patterns. The finding of TbGBP1 with a similar scaffold role to bridge TbSTING1 to TbSQSTM1 and TbLC3 to initiate autophagy and restrict HSV-1 production offers a unique window to understand the diverse regulatory roles of GBPs.
